# Carbon Nanotubes–Gr Inspired by Geckos’ Setae Structure with Enhanced Tribological Properties

**DOI:** 10.3390/ma18061221

**Published:** 2025-03-09

**Authors:** Jing Zhang, Yang Sun, Fengqin Shang, Zihan Yan, Jiayu Yao, Binghuan Chen, Hangyan Shen

**Affiliations:** 1 College of Materials and Chemistry, China Jiliang University, Hangzhou 310018, China; p22050854065@cjlu.edu.cn (J.Z.); p22050854042@cjlu.edu.cn (F.S.); s23050805024@cjlu.edu.cn (Z.Y.); p23050854056@cjlu.edu.cn (J.Y.); p24050858001@cjlu.edu.cn (B.C.); 2 School of Materials Science and Engineering, Tianjin University, Tianjin 300354, China

**Keywords:** setae structure, CNTs-Gr, microwave technology, tribological properties, lubricating film

## Abstract

The setae structure of geckos’ toes can create a strong adhesion force, allowing geckos to climb almost vertical walls. Inspired by this, carbon nanotubes–graphite (CNTs-Gr) was prepared by microwave technology, where CNTs like the setae structure grew in situ on the surface of Gr flakes. Compared to the Gr, the coefficient of friction (COF) and wear rate of CNTs-Gr decreased by 44% and 46%, reaching 0.10 and 1.18 × 10^−5^ mm^3^·N^−1^·m^−1^, respectively. Even if the load increased from 5 N to 35 N, the CNTs-Gr maintained a low and stable COF of 0.12. The excellent tribological properties were attributed to the unique setae structure of CNTs-Gr. This structure enabled the adhesion force of CNTs-Gr to the worn surface to increase threefold, improving the coverage of the lubricating film and significantly enhancing the lubricating film’s pressure resistance. The gecko setae structure proposed in this article provides researchers with a new idea for designing lubricants with excellent lubrication performance and high load-bearing capacity.

## 1. Introduction

Friction and wear are the main causes of energy loss and mechanical failure [1,2]. Adding lubricating materials is one of the most effective ways to reduce friction and wear, which is significant for energy conservation and prolonging mechanical life [3,4]. Common lubricating materials, such as graphite (Gr) [5,6,7], carbon nanotubes (CNTs) [8,9,10], MoS_2_ [11], etc., could form a lubricating film to isolate the contact between friction pairs, which is the key to reducing friction and wear [12].

Researchers have found that the area of lubricating film on the worn surface is an important factor affecting friction and wear performance. Xiao et al. [11] studied the friction properties of Cu-MoS_2_ composites and observed that expanding the coverage area of the MoS_2_ film on the worn surface from 0% to 70% resulted in a decrease in the COF from 0.76 to 0.18. The reduction in the COF could be attributed to the MoS_2_ lubricating film, which reduced the direct contact between the friction pairs. Its layered structure also provided excellent shear properties, further contributing to the reduction in friction. Similarly, Sun et al. [13] found that the Ag shell/Cu core structure effectively prevented an interfacial reaction between the Cu core and WS_2_ during hot-press sintering. As a result, the area of WS_2_ lubricating film on the worn surface of (Ag shell/Cu core)-WS_2_ composites was 64% higher than that on Cu-WS_2_ composites, resulting in a decrease in COF and wear rate by 66% and 79%, respectively. Therefore, increasing the area of lubricating film was an effective measure to decrease friction and wear. To increase the lubricating film area, Wei et al. [14] grafted chitosan-based copolymers onto the surface of graphene oxide, which could quickly and firmly adhere to the worn surface, significantly increasing the area of the lubricating film by 28%. Wang et al. [15] prepared silver-doped carbon quantum dots (Ag-CQDs) through the hydrothermal method and found that Ag-CQDs could adsorb onto the worn surface through chemical reactions with the substrates, resulting in a 9% increase in the area of the lubricating film to carbon quantum dots (CQDs). The lubrication performance of Ag-CQDs was significantly improved, with a reduction of 12% in COF and 8% in wear rate, respectively. The lubricating mechanism was attributed to the formation of a lubricating film composed of Ag-CQDs, multicomponent oxides, carbonates and nitrides, induced by tribochemical reactions, which provided effective protection for the worn surface and maintained a low COF and wear. Thus, enhancing the adhesion between lubricating materials and the worn surface was significant for improving the friction and wear properties. Although the grafting and hydrothermal methods used in the above works achieved success in enhancing the adhesion performance of lubricants, their preparation processes required lengthy synthesis steps and harsh reaction conditions, as well as the use of toxic catalysts and organic solvents [16,17]. Therefore, an efficient, convenient, and pollution-free method is needed to prepare lubricants with high adhesion performance.

**Figure 1:**
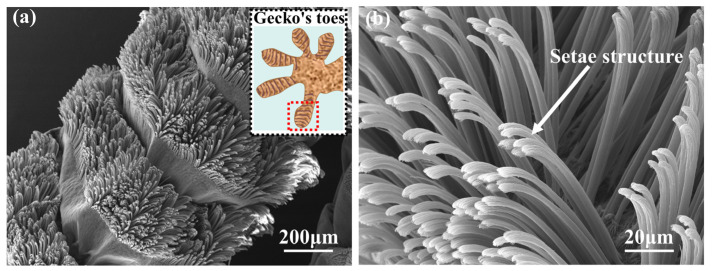
(**a**) Setae structure on a single toe of a gecko; (**b**) locally enlarged view of the setae structure. ((**a**) was the magnified image of the area within the red box).

Nature has provided excellent inspiration for designing and fabricating functional materials [18], such as shark skin swimsuits [19], imitation of lotus leaf hydrophobic films [20], and imitating spider web capture structures [21]. Gecko toes are also a common inspiration for building functional materials. Geckos can crawl lightly on glass surfaces thanks to the setae structure on their feet, as shown in Figure 1. This unique multi-stage setae structure can form good contact with the substrate surface and increase adhesion [22,23]. Typically, the gecko’s adult body weight is 50 to 100 g, and a gecko’s single foot can produce an adhesion force of 100 times gravity [24]. Ge et al. [25] transferred carbon nanotube arrays to flexible polymer ribbons to simulate setae structures to obtain four times the shear force of gecko feet. Therefore, constructing a gecko-like setae structure on the surface of lubricating materials improved their adhesion, and their lubrication performance was expected to be enhanced.

In this study, inspired by the gecko setae structure, microwave technology was used to grow CNTs on the surface of Gr flakes, with the CNTs growing in situ on the surface of the Gr flakes, resembling the setae structure of a gecko. Microwave technology was used to utilize the microwave absorption properties of Gr to rapidly, efficiently, and without pollution grow CNTs in situ. The tribological properties of CNTs-Gr were comprehensively studied using a ball-disk friction testing machine, the microstructure of the worn surface was characterized, and the lubrication mechanism of CNTs-Gr was explored. Specifically, the CNTs-Gr adhered to the worn surface through the strong adhesive force provided by the setae structure, forming a lubricating film with a large area, which reduced friction and wear.

## 2. Materials and Methods

### 2.1. Materials

Gr powder (99% purity, 6.5 μm), ferrocene (99% purity), and Naphthalene (99% purity) power were selected as the raw materials for preparing the CNTs-Gr. Gr and ferrocene power were purchased from Shanghai Aladdin Biochemical Technology Co., Ltd (Shanghai, China). Naphthalene power was purchased from Shanghai McLean Biochemical Technology Co., Ltd (Shanghai, China). CNTs were selected as the lubricant additives and were purchased from Nanjing Xianfeng Nanomaterials Technology Co. (Nanjing, China). Polyvinylpyrrolidone (PVP, 99% purity) was selected as the dispersant in the water-based lubricant and was purchased from Shanghai McLean Biochemical Technology Co., Ltd (Shanghai, China). All reagents were analytically pure.

### 2.2. Preparation of CNTs-Gr

**Figure 2:**
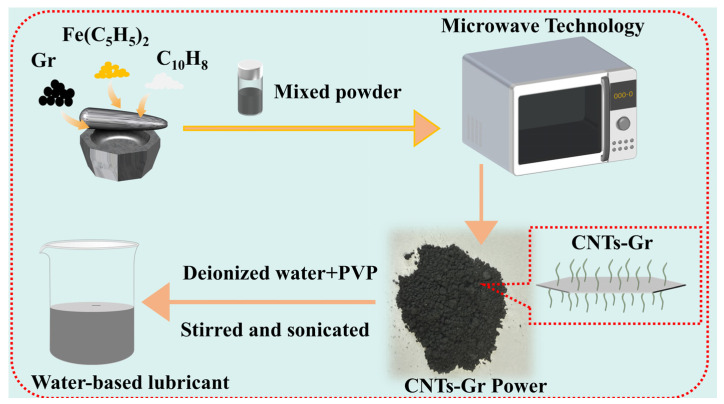
Schematic of the preparation process for CNTs-Gr.

The preparation process of the CNTs-Gr is illustrated in Figure 2. Firstly, Gr (200 mg), ferrocene (100 mg), and naphthalene (100 mg) were placed into an agate mortar and ground for 10 min. Next, the mixed powder was placed into a sealed quartz bottle and microwaved at 800 W power for 1 min. Then, the microwave-treated powder was ground with ferrocene (50 mg) and naphthalene (50 mg), followed by microwave treatment again at 800 W power for 1 min. Finally, the CNTs-Gr was successfully prepared by the above scheme.

### 2.3. Preparation of CNTs-Gr Water-Based Lubricant

Gr, CNTs, CNTs/Gr (the mechanical mixture CNTs and Gr, CNTs: Gr = 1:2), and CNTs-Gr were added as lubricant additives to the deionized water to prepare four different water-based lubricants, in which the concentration of all lubricant additives was 1.0 wt%. Then, PVP (0.1 wt%) was added as a dispersant to the water-based lubricants. Finally, all dispersions were stirred for 1 h using a magnetic stirrer and sonicated for 1 h at room temperature to obtain the uniformly mixed water-based lubricants, as shown in Figure 2.

### 2.4. Friction Test and Characterization

The friction and wear performance of the water-based lubricants was studied using a ball-disk friction testing machine (CSM Instrμments, Peseux, Switzerland) at room temperature. The top of the tester was a Si_3_N_4_ ball (Φ6 mm), and the disk was a polished stainless steel (SS) disk. During the friction process, both the SS disk and the Si_3_N_4_ ball were fully immersed in the prepared water-based lubricant. The entire friction experiment was conducted under submerged conditions in the water-based lubricant. Based on previous work and scientific references, the friction test was conducted under a load of 5 N at a speed of 0.5 cm/s for 15 min [26], and each experiment was repeated 3 times. After the friction test, the 3D profile of the wear track on the SS disk was determined utilizing a probe surface profiler (P-6, KLA-Tencor, Milpitas, CA, USA), which provided comprehensive data (cross-sectional profile, s) on the surface topography. The value of the wear volume (V) was calculated through V = s × l, in which l was the length of the wear track. The wear rate K of the worn surface was obtained by the following equation [27]:

(1) K=V/(F×L)

where *K* is the wear rate (mm^3^·N^−1^·m^−1^), *V* is the wear volume (mm^3^), *L* is the total sliding distance (m), and F is the normal load (5 N).

The morphology and structure of the CNTs-Gr were observed using a scanning electron microscope (SEM, SU8010, Hitachi Corporation, Tokyo, Japan), which allowed for high-resolution imaging of the sample surface and the identification of surface features at the micro- and nanoscale. The SEM could measure the elemental composition using an integrated Energy-Dispersive Spectroscopy (EDS) system, providing elemental maps and qualitative/quantitative analyses of the sample composition. The SEM equipped with EDS was used to analyze the elemental composition and distribution characteristics of the lubricating film. Transmission electron microscopy (TEM, TF20, FEI Company, Hillsboro, OR, USA) was used to study the lattice structure of CNTs-Gr at resolutions as low as the nanoscale. The phase structure of CNTs-Gr was characterized using an X-ray diffractometer (XRD, X’Pert3 Powder, PANalytical Company, Almelo, The Netherlands). The XRD technique provided phase identification and crystallographic information by measuring the diffraction pattern of X-rays that were scattered by the atomic planes in the sample. The XRD could operate over a 2θ range of 5° to 90° and could measure the lattice spacing and phase composition. However, a limitation of XRD was that it primarily analyzed the crystalline structures and might not have detected amorphous or poorly crystallized regions of the sample. A 3D laser scanning microscope (KEYENCE, VK-X1050, Osaka, Japan) was utilized to observe the worn surface on the SS disk. The 3D laser microscope provided precise height measurements and surface roughness mapping at the micro- and nanoscales. A difficulty during measurement was the surface preparation, as any contamination or roughness on the SS disk could have affected the accuracy of the worn surface measurement.

## 3. Discussion

### 3.1. Characterization of CNTs-Gr

**Figure 3:**
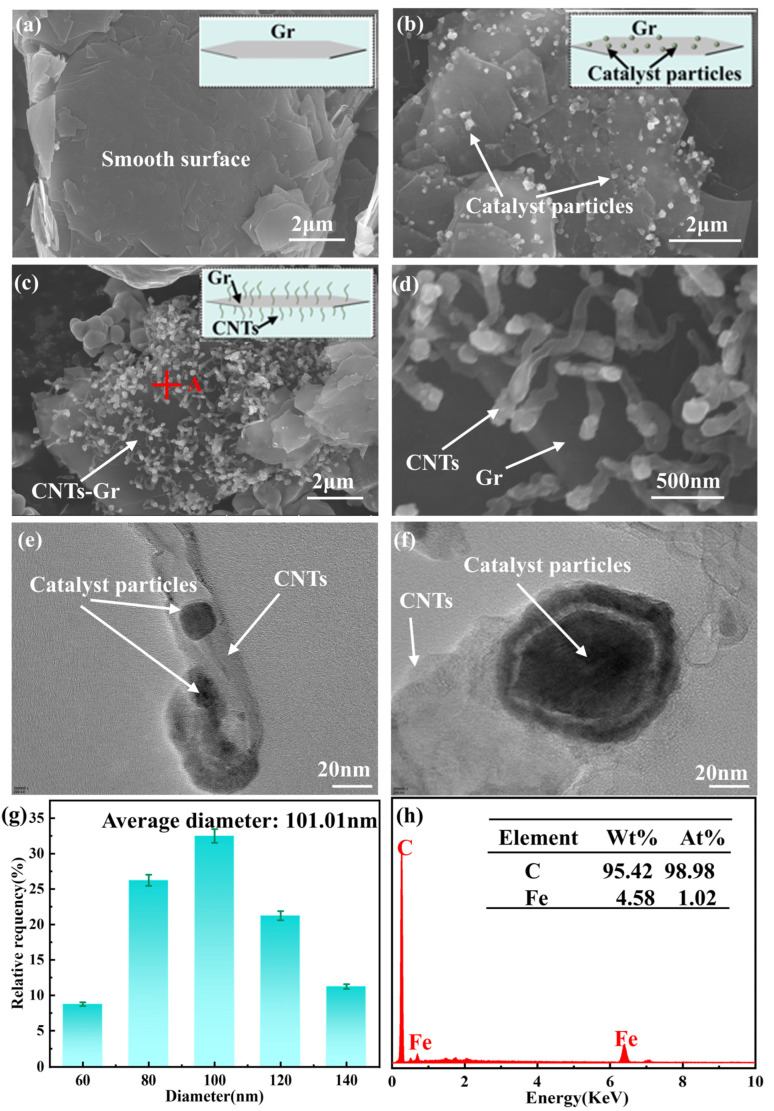
SEM images of (**a**) Gr and (**b**–**d**) CNTs-Gr; TEM images of (**e**,**f**) CNTs in CNTs-Gr; (**g**) diameter distribution of CNTs; and (**h**) EDS spectra of CNTs-Gr. (The letters and symbols in (**c**) represent the location of the EDS).

Figure 3 presents the microstructures of Gr and CNTs-Gr, which were synthesized through microwave technology. It can be seen in Figure 3a that the Gr showed a typical sheet-like structure, the surface of which was very smooth. According to the process shown in Figure 2, when the mixture of Gr, ferrocene, and naphthalene was heated by microwave technology, the ferrocene decomposed into nanocatalyst particles and anchored onto the Gr surface, as shown in Figure 3b. Then, these nanocatalyst particles absorbed the carbon source provided by naphthalene decomposition and promoted the growth of CNTs through the tip growth mechanism [28]. As a result, CNTs-Gr was formed, as shown in Figure 3c, d, in which numerous CNTs grew along the smooth surface of the Gr flakes, creating a structure resembling gecko setae. Figure 3e,f are enlarged images of the top structure of CNTs in CNTs-Gr (Figure 3d), showing that the nearly circular nanocatalyst particles were completely wrapped in the walls of CNTs. The nanocatalytic particles continuously catalyzed the formation of CNTs, which were pushed upwards by the grown CNTs [28]. Furthermore, a vertically upward structure of CNTs was formed on the surface of the Gr. In addition, image analysis software (ImageJ 1.x) was used to analyze over 100 CNTs in the CNTs-Gr, where the average diameter of the CNTs was 101.01 nm, and their diameter distribution is shown in Figure 3g. The average length of the CNTs was 0.83 μm, and their distribution density on the Gr surface was 7.57/μm^2^. Figure 3h shows that the CNTs-Gr was composed of C and Fe elements, in which the C element content accounted for 95.42 wt%. The atomic percentage was analyzed, with C accounting for 98.98 at%, and Fe accounting for 1.02 at%. Among them, the Fe element was mainly provided by the catalyst particles from the decomposed ferrocene [28].

**Figure 4:**
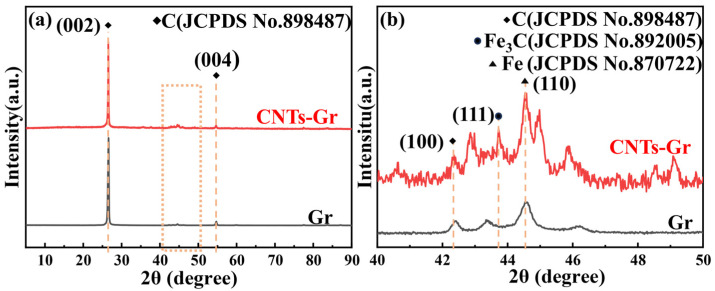
(**a**) XRD pattern of Gr and CNTs-Gr and (**b**) local enlarged pattern.

Figure 4 presents the XRD patterns of Gr and CNTs-Gr. The characteristic peaks of Gr and CNTs-Gr at 26.6°, 42.4°, and 54.6° could be attributed to the (002), (100), and (004) crystal planes of Gr (JCPDS No. 898487). In addition, unique peaks were observed in the XRD pattern of CNTs-Gr, with the peak at 43.7° corresponding to the (111) plane of carbide iron (Fe_3_C) and the peak at 44.6° corresponding to the (110) plane of iron (Fe) [29]. The results indicated that the nanocatalysts in Figure 3e were Fe and Fe_3_C. Prominent peaks in the XRD spectrum showed that the CNTs-Gr prepared by microwave technology were composed of CNTs, Gr, and catalyst particles (Fe_3_C, Fe).

### 3.2. Tribological Behavior of CNTs-Gr and Characterization of the Worn Surface

**Figure 5:**
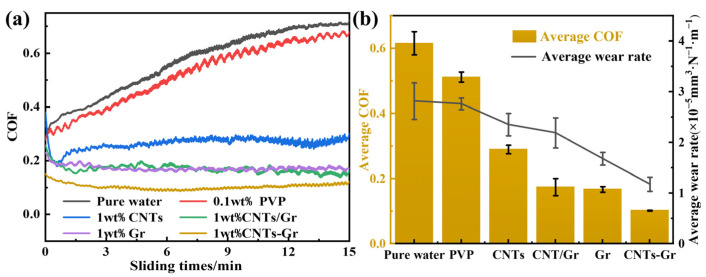
(**a**) The COF curves of pure water, PVP, CNTs, CNTs/Gr, Gr, and CNTs-Gr and (**b**) average COF and wear rate.

The COF and wear rate of the CNTs-Gr and control samples (Gr, CNTs, CNT/Gr) are presented in Figure 5. It can be observed from Figure 5 that the COF and wear rate of the pure water were the highest, which were 0.62 and 2.82 × 10^−5^ mm^3^·N^−1^·m^−1^, respectively. After adding PVP (0.1 wt%) to the pure water, the COF and wear rate were similar to those of pure water, indicating that PVP had little effect on the lubrication performance of pure water. However, when Gr and CNTs were added into water, the lubrication performance of pure water could be significantly improved. The COFs of the Gr and CNTs decreased to 0.17 and 0.32, respectively, and the wear rates decreased to 1.68 × 10^−5^ mm^3^·N^−1^·m^−1^ and 2.35 × 10^−5^ mm^3^·N^−1^·m^−1^, respectively. More interestingly, when CNTs-Gr was used as an additive, the COF and wear rate were the lowest, at 0.10 and 1.18 × 10^−5^ mm^3^·N^−1^·m^−1^, respectively. Compared to the Gr, there was a reduction of 44.1% in the COF and 46.1% in the wear rate. Therefore, the CNTs-Gr exhibited excellent tribological performance and possessed a lower COF and wear rate.

**Figure 6:**
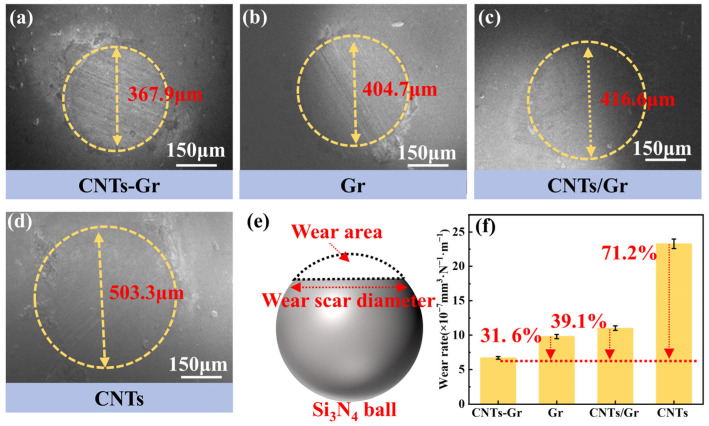
SEM images of the worn surfaces of the Si_3_N_4_ ball: (**a**) CNTs-Gr, (**b**) Gr, (**c**) CNTs/Gr, and (**d**) CNTs lubricant. (**e**) Calculation model and (**f**) wear rate of Si_3_N_4_ ball.

In order to obtain the wear condition of Si_3_N_4_ balls, the wear surface was characterized using SEM, as shown in Figure 6. The wear surface diameters were ordered from small to large as CNTs-Gr, Gr, CNTs/Gr, and CNTs. The wear surface diameter of CNTs-Gr was 367.9 μm, while that of CNTs was 503.3 μm, as shown in Figure 6a, d. The wear rate of the Si_3_N_4_ ball was calculated according to the model [30] shown in Figure 6e, and the result is shown in Figure 6f. Compared to Gr and CNTs, the wear rate of the CNTs-Gr decreased by 71.2% and 31.6 %, respectively. When CNTs-Gr was used as a lubricant, both Si_3_N_4_ balls and SS disks (as shown in Figure 5b) exhibited the lowest wear rate, confirming that CNTs-Gr had superior lubrication and anti-wear properties compared to Gr and CNT.

**Figure 7:**
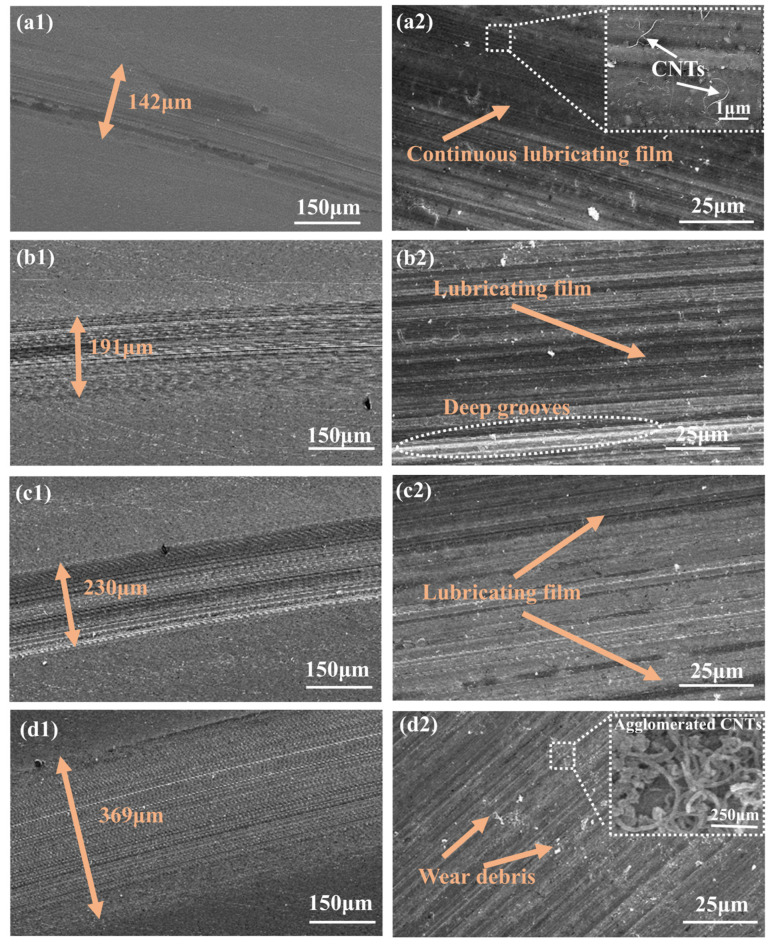
(**a1**,**a2**), (**b1**,**b2**), (**c1**,**c2**), and (**d1**,**d2**), respectively, depict the worn surfaces of SS disks when using CNTs-Gr, Gr, CNTs/Gr, and CNTs lubricant.

To investigate the lubrication mechanism, a detailed analysis was conducted on the worn surface of the SS disk. Figure 7(a1–d2) show the worn surfaces and their magnified images under lubrication of CNTs-Gr, Gr, CNTs/Gr, and CNTs, respectively. The minimum diameter of the worn surface with CNTs-Gr was 142 μm, representing reductions of 25.7% and 61.55% compared to Gr and CNTs, respectively. By magnifying the worn surface, it was found that the dark black area on the worn surface was the lubricating film. As shown in Figure 7(a2), a continuous lubricating film was present on the worn surface of the SS disk that was lubricated with CNTs-Gr, and under high-magnification SEM, CNTs were observed on the surface of the lubricating film. This indicated that the setae structure of CNTs-Gr could effectively adhere to the worn surface, forming a lubricating film with a large area. It was precisely this lubricating film with a large area that led to the reductions in the COF and wear rate [11,31]. In Figure 7(b2), it can be seen that Gr formed a partial lubricating film on the worn surface, and the worn surface exhibits deep grooves. In Figure 7(d2), it can be seen that the worn surface under the CNT lubricant shows almost no lubricating film and a large amount of wear debris, produced by friction. At the same time, aggregates of CNTs were also observed on the worn surface, which could lead to an increase in the COF and wear rate [32,33]. Therefore, CNTs-Gr was able to form a continuous and complete lubricating film structure on the worn surface, leading to reductions in COF and wear.

**Figure 8:**
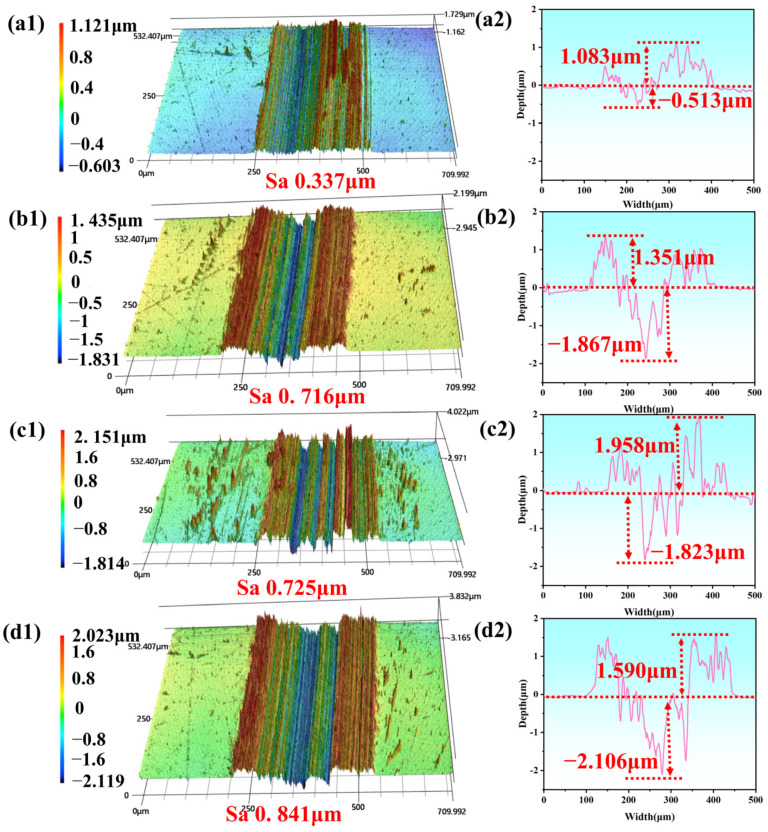
Three-dimensional profiles and cross-sectional wear scar curves of SS with different lubricant additives. (**a1,a2**) CNTs-Gr, (**b1,b2**) Gr, (**c1,c2**) CNTs/Gr, and (**d1,d2**) CNTs.

A 3D laser scanning microscope was further used to evaluate the worn surfaces of the SS disk. Figure 8 presents the 3D profiles and cross-sectional wear scar curves of the worn surfaces under the four lubricant additives of CNTs-Gr, Gr, CNTs/Gr, and CNTs. As shown in Figure 8(d1,d2), the 3D profiles of CNTs were severely serrated, and the height of the worn surface burr was very irregular. In Figure 8(c1,c2), with the addition of CNTs/Gr, the sawtooth phenomenon is weakened and the irregularity of the burr height is reduced. Figure 8(a1,a2) shows that the serrated shape of the 3D profiles of CNTs-Gr was the least obvious, which indicated that the roughness of its worn surface was significantly reduced. This was due to the formation of a continuous lubricating film structure on the surface by CNTs-Gr, as shown in Figure 7(a2). Furthermore, the surface roughness parameter Sa, calculated from the 3D profile, revealed that CNTs-Gr had the smallest Sa (0.337 μm), while CNTs exhibited the largest Sa (0.841 μm). The increase in the surface roughness of the worn surface led to an increase and fluctuation in the COF and an increase in the wear rate [31].

**Figure 9:**
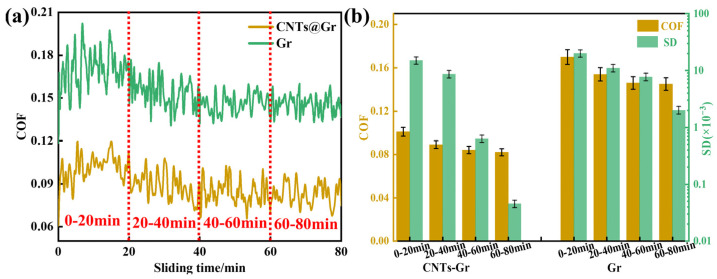
(**a**) The COF curves of Gr and CNTs-Gr; (**b**) average COF and SD every 20 min.

To compare the tribological properties of Gr and CNTs-Gr, the sliding time was increased to 80 min. Figure 9a shows the COF curves of the Gr and CNTs-Gr. Figure 9b shows the average COF and standard deviation (SD) every 20 min. The smooth COF curves represent a more stable tribological performance of the lubricating materials, and the SD was introduced to evaluate the stability [34,35]. In Figure 9b, it can be seen that at the sliding times of 0–20 min, the COF of CNTs-Gr was 0.10, with an SD of 15 × 10^−3^. As the sliding times increased, the COF and SD of CNTs-Gr gradually decreased to 0.08 and 0.05 × 10^−3^ (60–80 min), respectively. At the sliding times of 0–20 min, the COF of Gr was 0.17, with an SD of 20 × 10^−3^. As the sliding times increased, the COF and SD of Gr decreased to 0.15 and 2 × 10^−3^ (60–80 min), respectively. Compared to Gr, the CNTs-Gr exhibited lower COFs and SDs. The stable tribological performance of CNTs-Gr stemmed from CNTs-Gr adhering to the worn surface of the disk, thereby generating a lubricating film [36], which reduced the COF and SD [37].

**Figure 10:**
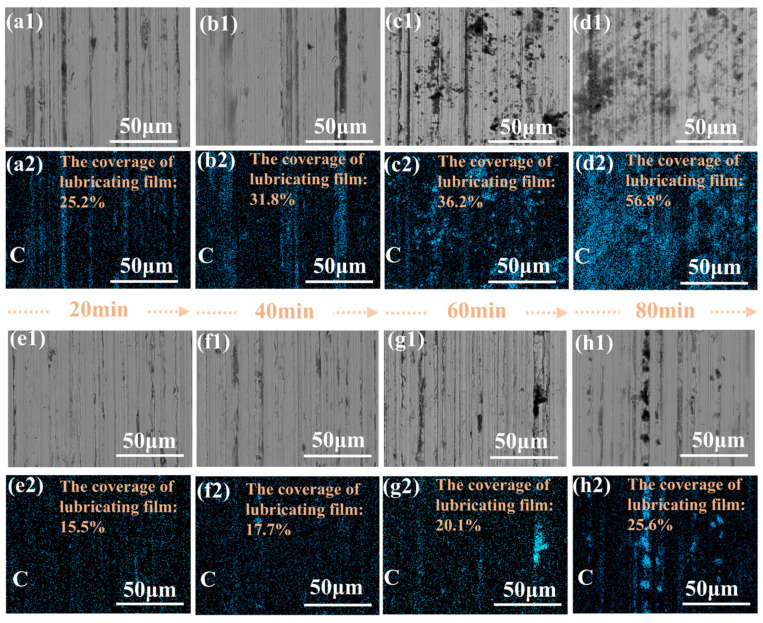
SEM and EDS images of worn surfaces on SS disks, lubricated with (**a1**–**d2**) CNTs-Gr and (**e1**–**h2**) Gr, within the sliding times 20, 40, 60, and 80 min.

Figure 10 shows the SEM and EDS images of the worn surface of the SS disk for CNTs-Gr (a–d) and Gr (e–h). Since the lubrication film was mainly composed of C, the coverage of the lubrication film on the worn surface could be confirmed by the C content, and the range rate of the lubricating film was statistically analyzed using image analysis software (ImageJ). The coverage of the lubricating film increased with the increase in the sliding times. At 20 min, the worn surfaces of CNTs-Gr and Gr were covered by 25.2% and 15.5% lubricating films, respectively, as shown in Figure 10(a2,e2). However, at 80 min, the coverage area of the CNTs-Gr lubricating film reached 56.8%, which was 121.9% higher than that under the action of Gr. The EDS results indicated that CNTs-Gr effectively adhered to the worn surface, forming a lubricating film with a large area, which reduced the COF and wear rate.

**Figure 11:**
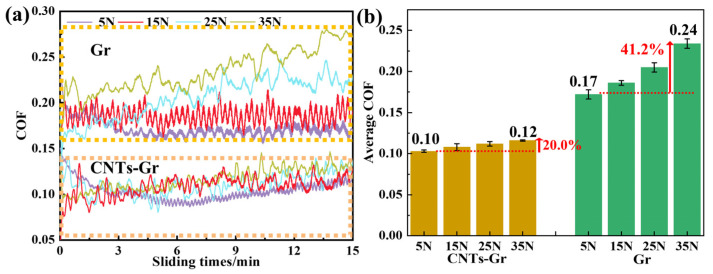
(**a**) The COF curves of Gr and CNTs-Gr under 5–35 N loads and (**b**) the average COF of Gr and CNTs-Gr under 5–35 N loads.

In Figure 11a, b, it can be seen that as the load increased from 5 N to 35 N, the COF of Gr rose by 41.2%. However, the COF of CNTs-Gr remained relatively stable under varying loads, with an increase of only 20.0% as the load went from 5 N to 35 N. This phenomenon was attributed to the setae structure of CNTs-Gr, which could still firmly adhere to the worn surface even under a 35 N load, ensuring that the lubricating film remained stable despite the increase in load.

**Figure 12:**
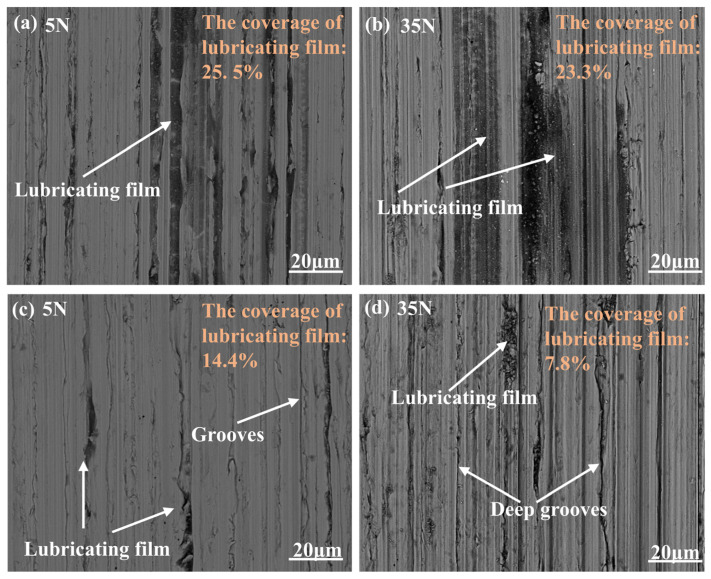
The SEM images of worn surfaces of CNTs-Gr lubricants under (**a**) 5 N and (**b**) 35 N and Gr lubricant under (**c**) 5 N and (**d**) 35 N.

Figure 12 shows the worn surfaces under Gr and CNTs-Gr lubrication at 5 N and 35 N loads. As shown in Figure 12a,c, the dark areas in the SEM images represented the regions covered by the lubricating film, and the range rate of the lubricating film was statistically analyzed using image analysis software (ImageJ). Under a 5 N load, the coverage of the lubricating film on the worn surface under CNTs-Gr lubrication was 25.5%. When the load increased to 35 N, the coverage of the lubricating film remained at 23.3%. Therefore, as the load increased, the COF of CNTs-Gr increased by only 20.0%, as shown in Figure 11b. In contrast, under a 5 N load, the coverage of the lubricating film on the worn surface under Gr lubrication was 14.4%, and when the load increased to 35 N, the coverage of the lubricating film decreased to only 7.8%, a reduction of 45.8%. As shown in Figure 12d, under Gr lubrication at 35 N, deep grooves appeared on the worn surface. This indicated that as the load increased, the lubricating film structure formed by Gr on the worn surface was largely destroyed, leading to a 41.2% increase in the COF, as shown in Figure 11b.

### 3.3. Analysis of Lubrication Mechanism

The above results indicated that CNTs-Gr could adsorb onto the worn surface and form a continuous lubricating film due to its setae structure. The adhesion force primarily originated from molecular interactions, whose strength was proportional to the contact area [38]. If the CNTs-Gr and SS substrates were regarded as two planes that were primarily attracted by van der Waals forces, the adhesion force at the contact interface could be approximated by the following equation [39]:

(2) $$F=HA/(6\pi d^{3})$$

where *H* is the Hamiltonian constant, *A* is the contact area, and d is the distance between the surfaces. Taking a typical *H* value of 0.4 × 10^−19^ J and d of about 1 nm [39], the contact area between the CNTs-Gr and the substrate (*A_CNTs-Gr_*) was the sum of the Gr’s contact area (*A_Gr_*) and the CNTs’ contact area (*A_CNTs_*), as shown in the following equation:

(3) $$A_{CNTs-Gr}=A_{Gr}+A_{CNTs}$$

**Figure 13:**
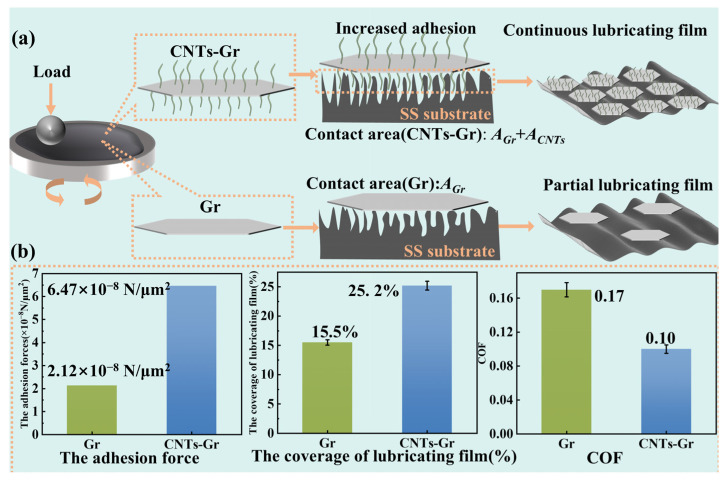
**(a)** Friction mechanism of CNTs-Gr additives; (**b**) The adhesion force, the coverage of lubricating film, and COF of Gr and CNTs-Gr.

The enhanced adhesion of CNTs-Gr was attributed to CNTs filling the voids of the substrate and increasing the contact area. Taking Gr as a plate and CNTs as a cylinder, it was assumed that the CNTs-Gr were in full contact with the substrate. The diameter of the CNTs was 101.01 nm, and the length was 0.83 μm, as determined by the image analysis software (ImageJ). The distribution density of CNTs on the Gr surface was 7.57/μm^2^. After the calculation, the contact area between 1 μm^2^ of CNTs-Gr and the substrate was 3.05 μm^2^. Using Equation (2), the adhesion forces of Gr and CNTs-Gr with the substrate were 2.12 × 10^−8^ N/μm^2^ and 6.47 × 10^−8^ N/μm^2^, respectively. Therefore, CNTs-Gr formed a continuous lubricating film structure, attributed to the strong adhesion force provided by its setae structure. The friction mechanism schematic of CNTs-Gr is shown in Figure 13. During the friction test, CNTs-Gr entered the friction area and increased the contact area with the worn surface through the setae structure, which improved the adhesion and formed the lubricating film. As the sliding proceeded, CNTs-Gr continued to adsorb on the worn surface, increasing the area of the lubricating film, which resulted in a gradual decrease in the COF to a stable value. Consequently, CNTs-Gr could avoid direct contact with the friction pair, providing efficient lubrication and anti-wear properties.

## 4. Conclusions

In this study, a new technology was proposed for the rapid synthesis of CNTs-Gr, and its microstructure and chemical properties were characterized in detail. In addition, the tribological properties and lubrication mechanism of the CNTs-Gr were systematically investigated. The prepared CNTs-Gr, due to its setae structure, exhibited excellent adhesion and could effectively adsorb onto the worn surface, forming a lubricating film with a large area. This structure significantly reduced the COF and wear, aligning with the anticipated performance improvements. The conclusions were as follows:

(1) CNTs-Gr was successfully prepared within 2 min using microwave technology. The CNTs in CNTs-Gr grew vertically and uniformly on the surface of Gr flakes, forming a gecko setae-like structure.

(2) The unique setae structure of CNTs-Gr led to an excellent lubrication performance. The COF and wear rate of the CNTs-Gr were 0.10 and 1.18 × 10^−5^ mm^3^·N^−1^·m^−1^, respectively. Compared to Gr, the COF and wear rate decreased by 44.1% and 46.1%, respectively. Even when the load was increased to 35 N, the COF of the CNTs-Gr remained stable at 0.12.

(3) The setae structure of CNTs-Gr tightly contacted the worn surface, generating strong adhesion (6.47 × 10^−8^ N/μm^2^) and promoting the formation of a lubricating film during the friction test. After 90 min of friction test, the lubricating film coverage reached up to 56.8%. Even under high loads, the lubricating film maintained good coverage.

(4) CNTs-Gr, with its excellent lubrication performance, could replace Gr as a lubricant in parts such as engines, brake systems, and wheel bearings. The preparation process only required a few minutes of microwave technology, thus not increasing the cost of use. Since the CNTs were grown in situ on the surface of Gr, this avoided the potential harm of nanoparticles to humans and the environment.

## Data Availability

The original contributions presented in this study are included in the article. Further inquiries can be directed to the corresponding authors.
